# Association between coenzyme Q_10_ and glucose transporter (GLUT1) deficiency

**DOI:** 10.1186/s12887-014-0284-5

**Published:** 2014-11-08

**Authors:** Delia Yubero, Mar O’Callaghan, Raquel Montero, Aida Ormazabal, Judith Armstrong, Carmina Espinos, Maria A Rodríguez, Cristina Jou, Esperanza Castejon, Maria A Aracil, Maria V Cascajo, Angela Gavilan, Paz Briones, Cecilia Jimenez-Mallebrera, Mercedes Pineda, Plácido Navas, Rafael Artuch

**Affiliations:** Clinical Biochemistry, Pediatric Neurology, Histopathology, Gastroenterology-Nutrition and Neuromuscular Unit Departments. Hospital Sant Joan de Déu and Centre For research in rare diseases (CIBERER), Instituto de Salud Carlos III, Passeig Sant Joan de Déu, 2, 08950 Esplugues, Barcelona Spain; Insituto de Investigación Príncipe Felipe, CIBERER, Valencia, Spain; Centro Andaluz de Biología del Desarrollo, Universidad Pablo de Olavide-CSIC-JA and CIBERER, Sevilla, Spain; Instituto de Bioquimica Clínica, Hospital Clinic i provincial, CIBERER, Barcelona, Spain

**Keywords:** Glucose transporter type I deficiency, *SLC2A1* gene, Coenzyme Q_10_, Ataxia, Ketogenic diet

## Abstract

**Background:**

It has been demonstrated that glucose transporter (GLUT1) deficiency in a mouse model causes a diminished cerebral lipid synthesis. This deficient lipid biosynthesis could contribute to secondary CoQ deficiency. We report here, for the first time an association between GLUT1 and coenzyme Q_10_ deficiency in a pediatric patient.

**Case presentation:**

We report a 15 year-old girl with truncal ataxia, nystagmus, dysarthria and myoclonic epilepsy as the main clinical features. Blood lactate and alanine values were increased, and coenzyme Q_10_ was deficient both in muscle and fibroblasts. Coenzyme Q_10_ supplementation was initiated, improving ataxia and nystagmus. Since dysarthria and myoclonic epilepsy persisted, a lumbar puncture was performed at 12 years of age disclosing diminished cerebrospinal glucose concentrations. Diagnosis of GLUT1 deficiency was confirmed by the presence of a *de novo* heterozygous variant (c.18+2T>G) in the *SLC2A1* gene. No mutations were found in coenzyme Q_10_ biosynthesis related genes. A ketogenic diet was initiated with an excellent clinical outcome. Functional studies in fibroblasts supported the potential pathogenicity of coenzyme Q_10_ deficiency in GLUT1 mutant cells when compared with controls.

**Conclusion:**

Our results suggest that coenzyme Q_10_ deficiency might be a new factor in the pathogenesis of G1D, although this deficiency needs to be confirmed in a larger group of G1D patients as well as in animal models. Although ketogenic diet seems to correct the clinical consequences of CoQ deficiency, adjuvant treatment with CoQ could be trialled in this condition if our findings are confirmed in further G1D patients.

## Background

GLUT1 deficiency syndrome (G1D) most often causes infantile-onset refractory epilepsy, cognitive impairment and motor abnormalities (ataxia, dystonia, chorea or dyskinesia) [1-4]. The main pathophysiological mechanism of the disease is associated with impaired glucose transport across the blood brain barrier and through astrocyte cell membranes that are haploinsufficient in the GLUT1 glucose carrier encoded by the *SLC2A1* gene [5,6]. However it is unclear how decreased glucose flux leads to the manifestations of the disorder.

G1D is a partially treatable condition with ketogenic diet (KD), which can replace glucose for acetyl-CoA generation [7,8]. Energy failure has been proven in G1D astrocytes, while tricarboxilyc acid abundance in the brain of G1D mouse model is normal. These findings support the complementary or alternative view that additional mechanisms participate in disease pathogenesis, placing new emphasis on G1D as a glial disease [9]. This contention is highlighted by the preliminary therapeutic efficacy of triheptanoin, a dietary supplement with the potential to stimulate cerebral anabolism and energy delivery [10].

Coenzyme Q_10_ (CoQ) is a lipidic electron carrier in the mitochondrial respiratory chain (MRC). One of the essential substrates for CoQ generation is acetyl-CoA [11]. Mutations in genes involved in the CoQ biosynthesis pathway are associated with different clinical phenotypes, being cerebellar ataxia the most common one [11]. However, “secondary” CoQ deficiency is also characteristic of other diseases, since CoQ biosynthesis involves an intricate, broad set of reactions that may be potentially impacted by “primary” disturbance of several biochemical processes [12].

Our aim is to describe, for the first time a relevant association between G1D and CoQ deficiency. Clinical, biochemical, molecular and therapeutic observations constitute the basis of this association.

## Case presentation

The proband is a 15-years old girl with an unremarkable family history. At 18 months old she manifested no ambulation, and by 3 years old she had developed ataxia and epilepsy with normal neuroimaging. At 5 years old clinical examination showed axial hypotonia, truncal ataxia, limb dysmetria and hyperreflexia. Paroxysmal nystagmus, saccadization of visual pursuit and dysarthria were observed. The patient also exhibited intellectual disability (IQ = 54).

Biochemical analyses in blood disclosed elevated lactate in 3 separate occasions and elevated alanine in 12 determinations (Table 1). Muscle and skin biopsies were analyzed at 8 years of age to search for mitochondrial disorders. CoQ deficiency was identified both in muscle and fibroblasts (see Results). Because of this finding, CoQ supplementation (orally administered at 30 mg/Kg/day) was initiated. Ataxia improved dramatically after 6 months of therapy, and, upon reassessment after 4 years of CoQ treatment, ambulation remained essentially normal, with a mild residual reduction in velocity. Her nystagmus had also disappeared and her visual pursuit had normalized [13]. However, mild dysmetria, dysarthria, myoclonic epilepsy and intellectual disability (perhaps refractory to CoQ), were present. During CoQ therapy, concomitant treatment with valproate and ethosuximide was given to control myoclonic epilepsy. No noticeable side-effects were observed when antiepileptic doses were raised to maintain therapeutic levels along the evolution of the disease due to the patient increasing weight. In order to further investigate these manifestations, a lumbar puncture was performed at 12 years of age, revealing diminished cerebrospinal glucose concentrations (Table 1). Plasma glucose concentration was normal. Diagnosis of GLUT1 deficiency (G1D) was established and a ketogenic diet (4:1 ratio, containing medium chain triglyceride oil) was initiated (CoQ treatment was then discontinued).

**Table 1:** **Main biochemical findings in a case with GLUT1 and CoQ deficiency**

	**Patient**	**Reference values**
**Blood**		
Lactate	1.8-2.6 (2.2)	0.5-1.7 mmol/L
Alanine	300-691 (560)	150-270 μmol/L
Cholesterol	4.0	< 5.2 mmol/L
CoQ (baseline)	0.33	0.45-1.1 μmol/L
CoQ (after treatment)	3.9-7.8 (5.5)	0.45-1.1 μmol/L
**Cerebrospinal fluid**		
Glucose	1.9	2.2-3.4 mmol/L
Lactate	1.07	1.1-2.2 mmol/L
**Muscle biopsy**		
CoQ content	76	115-450 nmol/g protein
Complex I + III*	149	107-560 mU/citrate synthase U
Complex II + III*	82	75-149 mU/citrate synthase U
**Fibroblast**		
CoQ content (incubated with glucose)	78	100-150 nmol/g protein
CoQ content (incubated with galactose)	123	100-150 nmol/g protein

Patient results from blood are expressed as range (median).

*Complex I + III: NADH:cytochrome C oxidoreductase. Complex II + III: succinate:cytochrome C oxidoreductase.

Written informed consent was obtained from the parents. The study was approved by the Ethical Committee of our Hospital.

### Laboratory investigations

Muscle and skin biopsies were collected, stored and cultured following previously reported procedures [14]. CoQ content was analyzed by HPLC with electrochemical detection, and MRC enzyme activities by spectrophotometry as reported [14].

Functional studies in fibroblasts: Human Dermal Fibroblasts (HDF) were grown in Dulbecco’s modified essential medium as previously reported [15]. Cells were grown with 1 g/L of either glucose or galactose from plating and throughout the entire duration of the observation period. Mycoplasma testing was negative. Also normal and G1D fibroblasts were supplemented with 30 μmol/L CoQ. Growth rate was analyzed as indicated [15].

Molecular analysis of genomic DNA (gDNA) isolated from blood included Sanger sequencing of 12 genes involved in CoQ biosynthesis (*ADCK3*, *ADCK4, PDSS1, PDSS2, COQ2, COQ3, COQ4, COQ5, COQ6, COQ9, CQ10A* and *COQ10B*), in addition to the *SLC2A1* gene, which encodes for GLUT1 transporter. *In silico* mutation analysis was done by the Mutation Taster software.

Total RNA was isolated from control and patient’s fibroblast cultures with RNeasy Fibrous Tissue mini kit (Qiagen, Hilden, Germany). 1 μg of RNA was retro-transcribed with GoTaq® Probe 2-Step for RT-PCR (Promega, Wisconsin, USA) to obtain cDNA. *SLC2A1* mRNA transcripts were studied on cDNA through PCR amplification with specific primers located in exon 1 (available on request), followed by direct sequencing.

## Results

**Figure 1:**
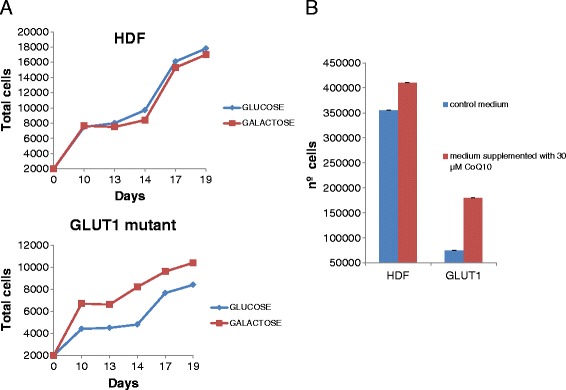
**Growth of GLUT1 fibroblast mutant cell line compared with control Human Dermal Fibroblasts (HDF). A)**. Both cells were seeded (2.000 cells/plate) and incubated with either glucose or galactose. **B)**. Both cells were seeded (50.000 cells/plate) and grown for one week with or without 30 μM CoQ_._

Muscle and fibroblasts CoQ content was decreased (Table 1). Functional studies of cell viability in culture with either glucose or galactose are illustrated in Figure 1A. The GLUT1 mutant cell line cultured in the presence of glucose displayed significantly decreased growth rate when compared with cultures grown in medium with galactose. Furthermore, the control cell line growth rate was significantly greater than those observed for the GLUT1 mutant line when cultured in glucose-containing medium. CoQ content was increased in GLUT1 mutant fibroblasts after 10 days growth in galactose (Table 1) while it remained unchanged in fibroblasts incubated in glucose media for the same length of time. CoQ supplementation of patient’s fibroblasts for one week induced an increase of 37% cell growth rate while the growth rate of control cells with the same treatment only increased by 15% (Figure 1B).

**Figure 2:**
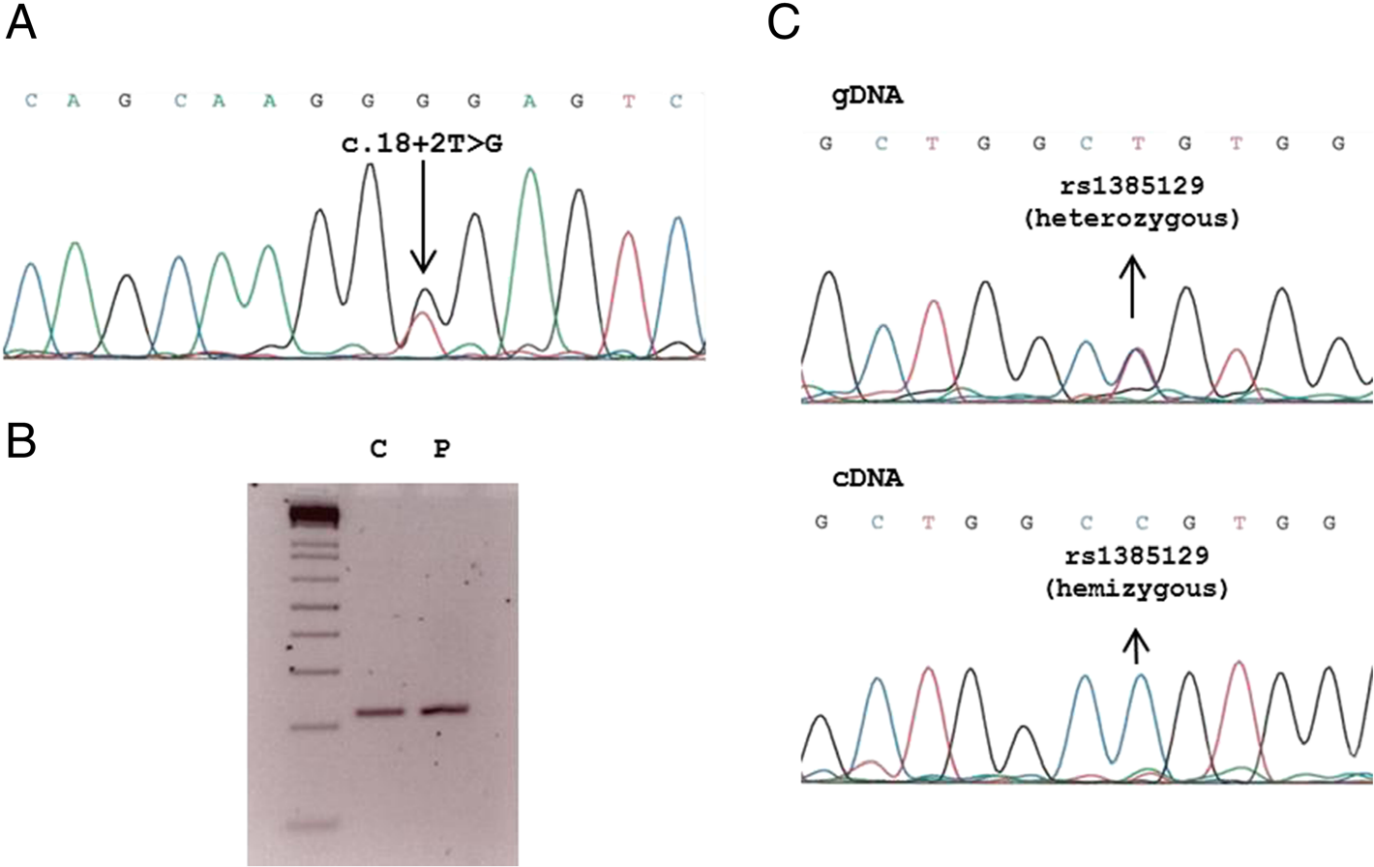
**Genetic analysis. A**- Electropherogram containing genomic *SLC2A1* DNA mutation in a case with GLUT1 deficiency. **B**- 3% agarose gel showing a 219 base pairs PCR amplification of *SLC2A1* cDNA region enconmpassing the spling mutation. C (control) and P (patient). **C**- Patient’s electropherograms showing a synonymous polymorphism (rs1385129, see dbSNP database) in heterozygous state in gDNA and in hemizygous state in cDNA.

Sequencing of CoQ-related genes disclosed no known pathogenic variants. G1D was established after the detection of a new heterozygous variant (c.18+2T>G) in the first intron of the *SLC2A1* gene (Figure 2A). The variant was not present in 200 studied alleles of Spanish ancestry. Conservation of the nucleotide position was high according to Phylophen scores and splice prediction software indicated a truncation of the splice site donor (data not shown). The cDNA region encompassing the genomic DNA mutation was amplified to detect a possible altered mRNA transcript in the patient. The PCR product did not show alternate amplification bands other than the control one (Figure 2B). Patient’s sample direct sequencing showed only the wild type transcript, lacking any hint of an aberrant transcript consequence of the intronic mutation identified in gDNA. Concurrently, patient carried a heterozygous SNP (rs1385129) located in exon 2, initially identified in gDNA (both alleles (T/C) were represented). We analyzed this SNP on the cDNA sequence (Figure 2C), and only one allele was present (C), pointing out that half doses of wild type mRNA was not present. Since the variant is located in the splice donor site and we only detected one allele of rs1385129 in the patient’s cDNA, downstream of c.18+2T>G, we hypothesize that splicing of the *SLC2A1* mRNA could be altered.

### Therapeutic interventions

After initiation of a ketogenic diet at 14 years old, dysarthria was controlled. At this age and prior to KD, mioclonic jerks with absences were present and EEG disclosed slow basal rhythm (alpha: 7–8 Hz) and generalized spike-wave discharges at 2.5 HZ, suggesting atypical absences. At that moment, patient was treated with valproate and ethosuximide. Epilepsy was totally controlled after 3 months of KD, and EEG disclosed normal results, except for slow basal activity. Antiepileptic treatment was progressively removed after 7 months of KD. At 15 years EEG showed a normal basal rhythm without paroxysms. Ataxia did not appear during KD therapy. Plasma CoQ levels were normal after initiation of a KD (0.58 μmol/L; reference values shown in Table 1).

## Discussion

We present for the first time, the identification of CoQ deficiency in a patient with G1D. Our clinical and biochemical observations warrant further discussion because of their potential pathophysiological and therapeutic implications.

Concerning the pathophysiology of G1D, Marin-Valencia et al. [9], demonstrated in a G1D mouse model a diminished cerebral lipid synthesis. Importantly, this deficient lipid biosynthesis could contribute to secondary CoQ deficiency. The muscle CoQ deficiency detected in our patient was mild, and this would explain that MRC enzyme activities were low-normal. However, we interrogated mitochondrial function due to the conjunction of the clinical findings and the consistent hyperlactacidemia and hyperalaninemia observed in our case. The experiments conducted in fibroblasts confirmed the CoQ deficiency, in agreement with its potential role in the pathophysiology of G1D [11]. Especially relevant were the differences of growth observed between fibroblasts cultured with either glucose or galactose and the recovery of CoQ in galactose growth. Glucose transport was impaired in the mutant line, but not galactose influx, which is able in turn to promote CoQ biosynthesis [15]. The human genome encodes for fourteen GLUT proteins that cooperate to transport different substrates other than glucose [16].

The normalization of growth of G1D cells after the incubation of fibroblasts with CoQ suggests the potential benefit of this coadjuvant therapy for G1D patients, which deserves further investigations. Although the mutation detected in *SLC2A1* is compatible with the common G1D phenotype, we cannot rule out the existence of mutations in other genes involved in CoQ metabolism given the fact that the CoQ metabolic pathway is not well understood.

The observed clinical improvement after CoQ supplementation in our patient supports these statements given that the most severe cerebellar manifestations proved amenable to CoQ therapy [13]. The simplest interpretation of our clinical observations is provided by the notion that the cerebellum is an extremely sensitive organ to oxidative stress and energy metabolic disorders, consistent with the near-universal demonstration of cerebellar manifestations even in cases of mild CoQ deficiency [11,13]. Once a ketogenic diet was initiated, the clinical outcome was excellent, with a complete cessation of epilepsy and other signs. We did not simultaneously treat the patient with CoQ and the ketogenic diet in order to separately elucidate the individual effects of both interventions. It is possible that a ketogenic diet causes a recovery in lipid biosynthesis that favorably impacts CoQ abundance.

## Conclusions

CoQ deficiency might be a new factor in the pathogenesis of G1D, although CoQ deficiency needs to be confirmed in a larger group of G1D patients as well as in animal models. Although KD seems to correct the clinical consequences of CoQ deficiency, adjuvant treatment with CoQ could be trialled in this condition if our findings are confirmed in further G1D patients. Furthermore, since molecular basis of CoQ deficiency syndrome remains elusive in most cases, the investigation of GLUT1 deficiency is advisable in cases presenting ataxia and epilepsy.

### Consent

Written informed consent was obtained from the patient’s parents for publication of this case report and any accompanying images. A copy of written consent is available for review by the Editor-in-Chief of this journal.

## Abbreviations

G1D: GLUT1 deficiency syndrome
CoQ: Coenzyme Q_10_
MRC: Mitochondrial respiratory chain
gDNA: Genomic DNA
HDF: Human Dermal Fibroblasts
KD: Ketogenic diet
